# An Enhanced Multiobjective Double Row Layout Model considering the Machine Breakdowns

**DOI:** 10.1155/2022/6289609

**Published:** 2022-08-28

**Authors:** Shengze Duan, Ling Kang

**Affiliations:** The State Key Laboratory of Mechanical Transmission, Chongqing University, Chongqing 400044, China

## Abstract

Machine breakdowns (MB) often occur, resulting in changes in the production layout and the material flow and causing a surge in production cost. Obviously, it is imperative to consider MB in workshop layout. Scholars mainly focus on the double row layout problem in an ideal nonfailure condition while scarcely considering MB mentioned above in the layout field. This paper proposes an enhanced multiobjective double row layout model considering MB (MDRLP-MB) to fill the research gaps. That model adds redundancy to reduce the losses caused by machine failure based on the prediction of breakdowns. In MDRLP-MB, machines with the same machine type have the same size and failure rate. Besides, this paper redefines the biobjectives of material handling costs (MHC) and the utilization ratio based on the feature of MDRLP-MB. To reduce the complexity of the problem, a decision rule is developed to reduce the alternative machine types. Furthermore, it revises the NSGA-II to solve the MDRLP-MB effectively. Results of numerical experiments and application cases show MDRLP-MB can obtain the optimal set of Pareto solutions in the case of increasing different numbers of redundancy considering MB to provide a more refined layout scheme for decision making.

## 1. Introduction

Facility layout is an essential part of the factory's production planning, which determines working areas such as machines, workstations, and service areas in a given space. According to statistics, 20% to 50% of production costs are attributed to MHC, and the suitable improvement in layout can decrease such costs by 10% to 30% [1]. Conversely, unreasonable facility layout (such as poor adjacency between the machine and the long transportation distance) will directly affect the production system and decrease its performance. This article is inspired by the cell production method. The real background for this article comes from a machine workshop, and the data used are real process parameters. When machine breakdowns occur in the workshop, it leads to the reduction of production or even shutdown of the workshop, the cost of workers, restarting the machine and possible loss of opportunity, etc. Especially for multivariety and small-batch unit production methods, punctuality is often required. Delayed delivery of several hours may cause huge loss of reputation. Therefore, machine breakdowns need to be taken into account in the layout problem.

Usually, the workshop layout is treated as a static layout and designed under general conditions, but there are many exceptional cases in production. Sethi and Sethi [2] introduced two types of uncertainties into the workshop, including internal interference (workshop failures, queue delays) and external changes (changes in product demand). Among them, workshop failures are the main reason for the delay in the production plan. Groenevelt et al. [3] demonstrated the impact of production interruptions on output. Jones and Sharp [4] emphasized that failure information, modeling, and planning should be carefully understood. That can provide powerful fault control capabilities. The downtime duration of failure type is studied by Moohialdin and Hadidi [5]. If MB is not considered when designing a layout, it may not maintain high efficiency in some emergencies, which will cause production interruption and lead to considerable losses to the factory. So, by identifying some MB that occurs during the production process, a more realistic layout can be constructed. Adding redundancy of critical machine on the shop floor is one maintenance strategy. Usually, enterprises adopt the strategy of normal maintenance and postfailure maintenance. Besides, some new theoretical methods have been used to help realize the control of workshop production [6–8]. However, for real production, the maintenance of faulty machine will increase the man-hours of workers without redundancy, resulting in a series of shortcomings such as prolonging the production cycle. These new theories and maintenance strategies cannot solve this problem without a suitable redundancy for the workshop. In fact, there are two benefits to adding redundancy on the workshop; on the one hand, redundancy can improve some material flow routes; on the other hand, it can maintain production when the machine fails. Based on the above considerations, this article chooses redundant machine as the strategy to deal with the occurrence of failure. This article focuses on the layout with MB; it uses redundancy to solve this problem, which means the damaged machine can be replaced by redundancy for processing tasks.

In terms of the number of targets to be investigated, many researchers classified the facility layout problem as a single objective problem and focus solely on minimizing MHC. However, the closeness rating, workshop area, safety, and so on are also essential criteria in facility layout problems (FLPs); such indicators (especially the workshop area) have a massive impact on the layout of the workshop. Hence, the layout problem is treated as the multiobjective optimization problem (MOOP) in some recent articles. Three additional indicators are used to measure the quality of the layout [9–11]: the distance, the closeness requirements among the departments, and the desired aspect ratio. Kheirkhah et al. [12] combined material handling system design and facility layout design into a dynamic facility layout problem (DFLP). Due to the high construction cost of some factories (e.g., clean room) [13], the workshop area is also considered as one of the goals. Zuo et al. [14] and Che et al. [15] explained the importance of workshop areas in production and presented a biobjective optimization model to balance the tradeoffs between space consumption and MHC. Liu et al. [16] sought to optimize the shop floor's utilization ratio, MHC, and adjacency value. Many factories begin to adopt fully automatic production lines, and it will no longer be extended to arrange layouts considering the production factors of workers. Therefore, our article does not involve the adjacency value, distance requirements, and other goals but focuses on the MHC and workshop utilization, following the method of Liu et al. [16] on the utilization ratio. On the one hand, the MHC is redefined and a more comprehensive layout is got. On the other hand, the model obtains the Pareto solutions in different situations according to the number of redundancy to get the most suitable layout.

There are many ways to solve MOOP, a multiobjective genetic algorithm is applied to get the Pareto solutions in [11], and the algorithm proposed is a kind of Pareto-based approach. For the problem of choosing the target weight for each target, Singh and Singh [17] proposed four methods to determine the target weight, which makes the multiobjective facility layout problem (mFLP) design process more objective and completely independent of the designer. An improved simulated annealing method was developed to solve the MOOP [18]. This article mainly analyzes the model and uses the excellent method to improve the solution. Although on some problems the newly proposed algorithm may be more efficient [19–22], it is difficult to prove their suitability for the special problems in this paper. So using a general and efficient metaheuristic method is a reasonable way. As a multiobjective solution method, NSGA2 was created by Deb et al. [23] firstly. It has been cited tens of thousands of times so far, which demonstrates its superiority in solving problems, so this paper uses it as the solution method.

The remaining chapters are organized as follows.  Section 2 reviews the relevant literature.  Section 3 describes a new mathematical model for MB based on a double row layout.  Section 4 shows the complexity of the model and gives a solution. In Section 5, a real-world case is used to prove the effectiveness of the model and randomly generate 9 cases to enrich this conclusion. Finally, Section 6 describes the conclusions and future outlook.

## 2. Related Literature

There is a considerable amount of literature and extensive research from the web of science on the production problems [24–27]. Hence, it is necessary to pay attention to those papers most related to our work, where keywords in these articles include replicas, double row layout, and machine breakdowns. This section first introduces the concept of redundancy. Secondly, it reviews some literature on DFLP. Machine breakdowns are the last part of the review.

### 2.1. Replicas in the Facility Layout Problem

Adding more machines in the workshop could balance the workload and reduce lead times [28]. Besides these benefits, Zhao and Wallace [29] pointed out that adding machines can reduce the MHC in the workshop and adopted a quadratic assignment model to describe the problem. Koltai and Stecke [30] allowed different machines to perform the same operation and provide a mathematical model for the path-independent analysis of flexible manufacturing system capabilities based on the concept of operation types. The integrated machine allocation and layout problem (IMALP) was proposed by Urban et al. [31]; they thought that each product flows between preselected machine types; machines belonging to the same machine type have different working capabilities. A two-stage method is proposed to solve this problem [32], the first stage determined the unit where the machine is located, and the flow between machines is distributed in the next phase. More recently, Gülsen et al. [33] proposed the concept of machine sharing and allowed different products to pass through the machines with the same function. They also believed that the product flow can be divided into several branches. This paper adopt the definitions of machine types in these articles, in this way, the material flow table is the relationship between machine types, and the specific flow between each machine is obtained by our model. In addition, this model defines redundancy as a machine that is increased after the capacity is met and believes that each machine belonging to the same type has a high degree of consistency (that is, having the same size and inherent availability).

### 2.2. The Double Row Layout Problem (DRLP)

The first integer programming model for double row layout is proposed by Chung and Tanchoco [34]. In the last ten years, many articles on the double row layout have emerged. Zuo et al. [14] used a set of optimization strategies, including multiobjective tabu search and linear programming, to obtain the Pareto solutions. Chae and Regan [35] modified the double layout model by introducing tighter constraints. Gülsen et al. [33] extended the DRLP literature by introducing machine type to the double row layout. They considered that each machine type has multiple copies of the same function and then used a two-stage approach to improve the layout. Firstly, they determine the absolute position of the machines in the workshop, in other words, the rows and columns where the machine is located. For another, the distance and flow between each machine are optimized by an iterative method, which splits the above variables into two subsets and iterates over them as LPs alternately until convergence. In our paper, it also divides the solution process into two steps. The first stage is similar to the paper to get the absolute location of the machines. Furthermore, the next stage is significantly different; the model firstly gets the best flow planning because of the assumption that material flow only chooses the shortest route (there are only a few combinations of the best flow route for each task) and then optimizes the distance.

### 2.3. Machine Breakdowns

Papers that consider MB in the layout are rare. Nevertheless, in the workshop scheduling problem, time-related factors are usually considered. In particular, many scholars have regarded MB as one of the factors affecting time. Liu et al. [36] considered workshops suffering from MB and offered two strategies. One is to provide new job scheduling, and the other is to help the system resume work. Al-Hinai and ElMekkawy [37] assumed that all breakdowns can be considered as one failure. On this basis, they proposed that the probability of the machine failing and breakdown duration was related to the machine's working time. In addition, they also thought the machine would resume its previous operation after the repair is completed. Xiong et al. [38] exploited the characteristics of MB and developed two robust representation methods. Specifically, the first method is based on the breakdown probability, while the second considered the duration of the failure and the location of the damaged machine. Therefore, MB occurs simultaneously on the plant in this research. This paper uses inherent availability instead of the breakdown probability and duration, and the inherent availability of the MB does not depend on the machine's working hours at this time but the material flows through different machine types during the whole production period. The reason of choosing MB as a fixed parameter is that facility layout is a planning problem. Usually, the layout will not be easily changed after the design is completed. All possible material flow routes are also planned in the initial program. Besides, managers are inclined to use inherent availability to measure machines performance instead of other kinds of noise in real life. Therefore, our model tends to consider the failure as a fixed value that occurs and solves the layout problem at one time.

### 2.4. Summary

Compared with other studies and articles, the innovations of this paper are as follows: (1) In the description of the failure situation, the inherent validity is used to evaluate the failure situation of the workshop. (2) A more reasonable way of using redundant machine is adopted; that is, only the machine that meets the maximum production capacity is used. Not all machines are used. (3) In the workshop layout problem, most of the redundant settings are used to optimize the material flow. However, another benefit of increasing the working performance of the system is not considered. This paper considers the superiority of redundancy in response to failures.

## 3. The Double Row Layout Reconfiguration Problem considering Machine Breakdowns

### 3.1. Problem Statement

This paper studies the double row layout considering machine breakdowns (MDRLP-MB). To solve the problem of MB, some machine types may require one or more redundancy. After some machine fails, the flow route through the machines will be redistributed. The layout planning considers the new material flows resulting from MB, calculating the inherent availability of the MHC in different failure cases of occurrence to obtain the total expected value. Model's input variables are multiple products (each product includes the required quantity and processing sequence) for each production period (regarding each change of machine location as a sign of a new production period). The objective of the article is to obtain the optimal set of Pareto solutions (minimizing MHC and maximizing the workshop utilization) by determining the type and the number of redundancy and the precise material flow of every product among different machines according to the processing sequence.

To better form the model, based on some articles [16, 33], assumptions are listed as follows:

1. One machine has the same capacity to process different products
2. The same type of machine has the same size, inherent availability, and capacity
3. To avoid discussing the scheduling problems that may arise in the production process, this paper assumes that, in a production period, the different types of products are not processed simultaneously, but in a separate batch
4. The maintenance time of machines does not overlap, and machines are considered to be put into use after repairing
5. The amortization or depreciation cost of the machine is not part of the model; only the improvement of the layout by adding redundancy is considered

### 3.2. The Multiobjective Double Row Layout Reconfiguration Model considering Machine Breakdowns

**Figure 1:**
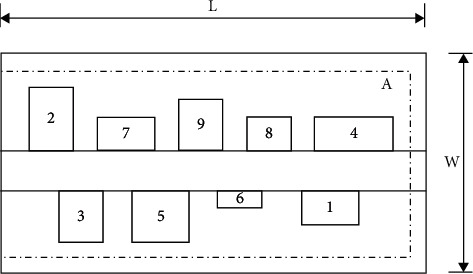
The double row layout problem.

This article adopts a double row layout model (DRLP), and the general layout is shown in Figure 1 (L, W, and A represent length, width, and area, respectively).

The bathtub curve reflects the working conditions of a single machine and is often used to describe the failure rate of the machine at different stages. This article assumes that the machine is in the stable wear period of the bathtub curve, where the mean time between failure (MTBF) is a fixed value, then counts the mean time to repair (MTTF), and gets an average value; then the inherent availability (*A*_*r*_) of machine *r* that is described by the following formula is also a fixed value.

(1) $$\begin{array}{c} A_{r}=\frac{\text{MTBF}}{\text{MTBF}+\text{MTTF}}. \end{array}$$

We define *z*_*k*_ as the cost for product *k* in the workshop, *z*_*rk*_ as the lowest MHC for product *k* after machine *r* is damaged, and *z*_0*k*_ ss the lowest MHC for product *k* without MB. According to the characteristics of workshop production, if only machine *r* is damaged, *z*_*k*_ can be expressed by

(2) $$\begin{array}{c} z_{k}=\left(1-A_{r}\right)*z_{rk}+A_{r}*z_{0k}+\left(1-A_{r}\right)*\frac{c-c_{r}}{c}*\left(z_{0k}+D*T\right), \end{array}$$

where *c* is the production line capacity without MB, *c*_*r*_ is the production line capacity when machine *r* fails, *D* is lost cost per unit time of delayed production, and *T* is total production time.

In two particular cases, *z*_*k*_ can be simplified to the following:

(1) The type of machine that can still meet the production capacity when a machine is damaged (i.e., own redundancy, *c*_*r*_=*c*):
  (3) $$\begin{array}{c} z_{k}=\left(1-A_{r}\right)*z_{rk}+A_{r}*z_{0k}. \end{array}$$
(2) The machine type which has only one machine (*c*_*r*_=0, in this case, *z*_*rk*_ becomes 0, which means that the workshop stops production after *r* is damaged and no material handling costs are incurred).

(4) $$\begin{array}{c} z_{k}=z_{0k}+\left(1-A_{r}\right)*D*T. \end{array}$$

To describe the mathematical model, notations are listed as follows:

 
*N0*: the number of machines to meet maximum capacity
 
*N*: the number of all machines (include redundancy) or locations
 
*M*: the number of products
 
*i*, *j*, *r* : indexes for machines(*i*, *j*=1,2,…, *N*); *i* ≠ *j*
 
*k*  : indexes for products(*k*=1,2,…, *M*)
 
*l*, *l*′ : indexes for machine locations(*l*, *l*′=1,2,…, *N*); *l* ≠ *l*′
 
*X*_*i*_: horizontal coordinate of machine *i*
 
*I*: expenditure of a unit material flow per unit distance
 
*f*_*rkij*_: when the machine *r* is damaged, the material flow of product *k* between machine *i* and machine *j*
 
*f*_*kij*_: the material flow of product *k* between machine *i* and machine *j* without MB
 
*d*_*ij*_: distance between machine *i* and machine *j*
 
*L*: the horizontal length of the workshop
 
*W*: the longitudinal length of the workshop
 
*C*: corridor width between two rows
 
*L*_*i*_: the horizontal length of machine *i*
 
*w*_*i*_: the longitudinal length of machine *i*
 
Δ*l*: the minimum horizontal distance between machines and workshop
 
Δ*l*_*ij*_: the minimum horizontal distance between machine *i* and machine *j*
 
Δ*w*: the minimum longitudinal distance between machines and workshop
 
Δ*k*_*i*_: the net clearance between machine *i* and the machine on its left (or the net clearance between the leftmost machine and the wall)
 
*X*_*il*_: if machine *i* is in position *l, X*_*il*_ = 1 and 0 otherwise
 
*q*_*il*_: if the machines *i* and *j* are in opposite rows, *q*_*il*_=0 and 1 otherwise
 
*p*_*rk*_: if the product *k* is processed on a machine *r,p*_*rk*_=1 and 0 otherwise
 
*S*_*i*_: area of machine *i*
 
*S*: the smallest rectangle that envelops all deployed machines
 
*c*: the production line capacity without MB
 
*c*_*r*_: the production line capacity when machine *r* fails
 
*D*: lost cost per unit time of delayed production
 
*T*: total production time
 
*z*_*rk*_: the lowest MHC for product *k* after machine *r* is damaged (if *c*_*r*_=0, *z*_*rk*_=0)
 
*z*_0*k*_: the lowest MHC for product *k* without MB
 
*A*_*r*_: inherent availability of machine *r*

Objective functions:

(1) Minimize
  (5) $$\begin{array}{c} f_{1}\left(x\right)=\sum_{k=1}^{M}\left(\sum_{r=1}^{N0}\left(1-A_{r}\right)*\left(z_{rk}+\frac{c-c_{r}}{c}*D*T\right)*p_{rk}\right.+\left.\left(1-\sum_{r=1}^{N0}\left(1-A_{r}\right)*\frac{c_{r}}{c}*p_{rk}\right)*z_{0k}\right). \end{array}$$
(2) Maximize

(6) $$\begin{array}{c} f_{2}\left(x\right)=\frac{\sum_{i=1}^{N}S_{i}}{S}. \end{array}$$

Subject to

(7) $$\begin{array}{c} \sum_{l=1}^{N}X_{il}=1,\;i=1,2,\ldots ,N, \end{array}$$

(8) $$\begin{array}{c} \sum_{i=1}^{N}X_{il}=1,\;l=1,2,\ldots ,N, \end{array}$$

(9) $$\begin{array}{c} \left|X_{i}-X_{j}\right|=\frac{l_{i}+l_{j}}{2}+\Delta l_{ij}+\Delta k_{i},\;i=1,2,\ldots ,N,\;j=1,2,\ldots ,N, \end{array}$$

(10) $$\begin{array}{c} \frac{l_{i}}{2}+\Delta l\leq X_{i}\leq L-\frac{l_{i}}{2}-\Delta l,\;i=1,2,\ldots ,N, \end{array}$$

(11) $$\begin{array}{c} w_{i}+\frac{C}{2}+\Delta w\leq \frac{W}{2},\;i=1,2,\ldots ,N, \end{array}$$

(12) $$\begin{array}{c} d_{ij}=\left|X_{i}-X_{j}\right|+C\left(1-q_{il}\right), \end{array}$$

(13) $$\begin{array}{c} \Delta l,\Delta l_{ij},\Delta w,\Delta k_{i}\geq 0, \end{array}$$

(14) $$\begin{array}{c} X_{il}\in \left\{0,1\right\},\;i=1,2,\ldots ,N,l=1,2,\ldots ,N, \end{array}$$

(15) $$\begin{array}{c} q_{il}\in \left\{0,1\right\},\;i=1,2,\ldots ,N,l=1,2,\ldots ,N, \end{array}$$

(16) $$\begin{array}{c} p_{rk}\in \left\{0,1\right\},\;r=1,2,\ldots ,N0,k=1,2,\ldots ,M, \end{array}$$

(17) $$\begin{array}{c} z_{rk}=\text{min}\left(\sum_{i=1}^{N0}\sum_{j=1}^{N0}\sum_{l=1}^{N0}\sum_{l^{\prime }=1}^{N0}\left({\text{If}}_{rkij}d_{ij}X_{il}X_{jl^{\prime }}\right)\right), \end{array}$$

(18) $$\begin{array}{c} z_{0k}=\text{min}\left(\sum_{i=1}^{N0}\sum_{j=1}^{N0}\sum_{l=1}^{N0}\sum_{l\prime =1}^{N0}\left(If_{kij}d_{ij}X_{il}X_{jl\prime }\right)\right). \end{array}$$

The objective function includes objective (5) and objective (6), the subobjective (5) is to minimize the total cost for one production period, this function can add a term which is the amortization (or depreciation) cost of redundant machines. The subobjective in (6) maximizes the workshop utilization. Constraint (7) guarantees that there is only one machine at each location. Each machine only occupies one location that is limited by constraint (8). Equation (9) represents the calculation method of the distance between two adjacent machines, that is, adding the net clearance while keeping the minimum clearance. Constraint (10) indicates the boundary constraint of the facility in the horizontal direction to ensure that it does not exceed the horizontal boundary. Constraint (11) represents the vertical boundary constraints of the facility to ensure that it does not exceed the vertical boundary. Equation (12) calculates the distance between two machines. Constraint (13) indicates that all distance constraints are positive. Equations (14)–(16) represent three binary variables. Equation (17) is the minimum MHC in the case of machine *r* damage. Equation (18) is the minimum MHC without MB.

**Figure 2:**
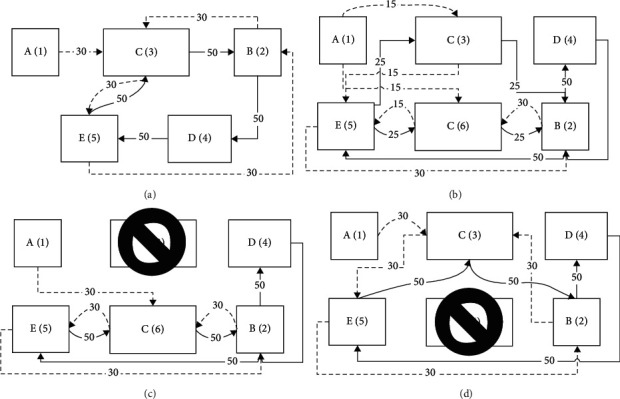
Example of machine breakdowns. (a) Initial layout. (b) Add replicas. (c) Breakdown condition 1. (d) Breakdown condition 2.

To illustrate the model mentioned above, this subsection depicts a simple case in Figure 2. In this case, there are two products in a certain production period, and there are five machine types in the workshop {A, B, C, D, E}, where the quantity of product 1 = 30, product 2 = 50. These two products will enter the workshop in a different batch, and the sequence of machine types passed by the product is as follows:

  Product 1: A-C-E-B-C is indicated by a dotted line in the figure
  Product 2: C-B-D-E-C is represented by a solid line in the figure

The inherent availability of the five machine types is {*A*_1_,  *A*_2_, *A*_3_,  *A*_4_,  *A*_5_}. If the layout in Figure 2(a) is adopted, there is only one machine per machine type, and the material flow between the machines will be fixed; the optimal layout is also easy to obtain through metaheuristics. Figure 2(a) is the best layout for this situation, and the material flows are as follows:

  Product 1: 1-3-5-2-3
  Product 2: 3-2-4-5-3

Now add a copy of type C to the layout of Figure 2(a) and name it C(6). Then the total expected cost considering the MB situation is using (17).

Now suppose that the optimal layout is obtained by the new model in Figure 2(b), and the MHC of the layout at this time is *z*_0_. It can be seen that, after adding a machine of C(6), the flow from A to C forms two branches, half of which flows to the upper side of C(3), the other half flows to the lower side of C(6), and then the two branches both enter the machine E(5). Finally, from E(5) flow to the next machine, that is, two flow routes 1-3-5-2-6 and 1-6-5-2-6 are formed. The flow route of product 2 is also formed in two branches 3-2-4-5-3 and 6-2-4-5-6.

By adding copies, not only is the material flow route changed, but also the reliability of the system is increased. For example, as shown in Figure 2(c) and Figure 2(d), since C(3) and C(6) belong to the same machine type, it is not necessary to stop the entire production line for repairing when one of them is damaged, just change the flow route to allow the processing to continue, and the flow routes of product 1 and product 2 will be regenerated. If the C(3) at the top of Figure 2(c) is damaged, the flow routes of product 1 and product 2 are 1-6-5-2-6 and 6-2-4-5-6, respectively. This article assumes that all flow passes through the above layout, and the costs incurred are recorded as *z*_3_, and then the MHC, in this case, is (1 − *A*_3_)*∗z*_3_+*A*_3_*∗z*_0_. Similarly, when the C(6) at the bottom of Figure 2(d) is damaged, the flow routes are 1-3-5-2-3 and 3-2-4-5-3, respectively, and the MHC is (1 − *A*_6_)*∗z*_6_+*A*_6_*∗z*_0_.

## 4. Characterizing MDRLR-MB Solutions

### 4.1. Complexity Analysis

The variables involved in the model include the selected machine types and number of copies, the relative and absolute position of the machines in the layout, and the possible production diversion caused by multiple redundancies. Firstly, set *n* as the number of candidate machine types and the addition of redundancy is *k*.

We can equate this problem with putting *k* balls into N boxes, so the number of ways that can add redundancy is given by

(19) $$\begin{array}{c} C_{n+k-1}^{k-1}. \end{array}$$

When the number of machine types is 10 and 5 redundancies are added, 1001 schemes will be generated, so it is not difficult to draw the following conclusions:

1. Adding redundancy will increase the number of machines involved in the layout, resulting in a sharp increase in the complexity of the problem. To simplify the complexity of the problem, use a simple decision rule to select the candidate machine type after solving the real-world case in chapter 5.
2. The problem complexity surges when the number of redundancy continues to increase, and the time required to solve the problem will increase significantly.

### 4.2. Solving MDRLR-MB with NSGA-II

As it is well known in literature, facility layout is an NP-hard problem. When there are too many machines, the computational complexity prevents us from using exact methods to obtain the solution set. Therefore, it is necessary to use metaheuristics to model and solve the problem. This article will use the classic NSGA-II algorithm to deal with the MDRLR-MB.

**Figure 3:**
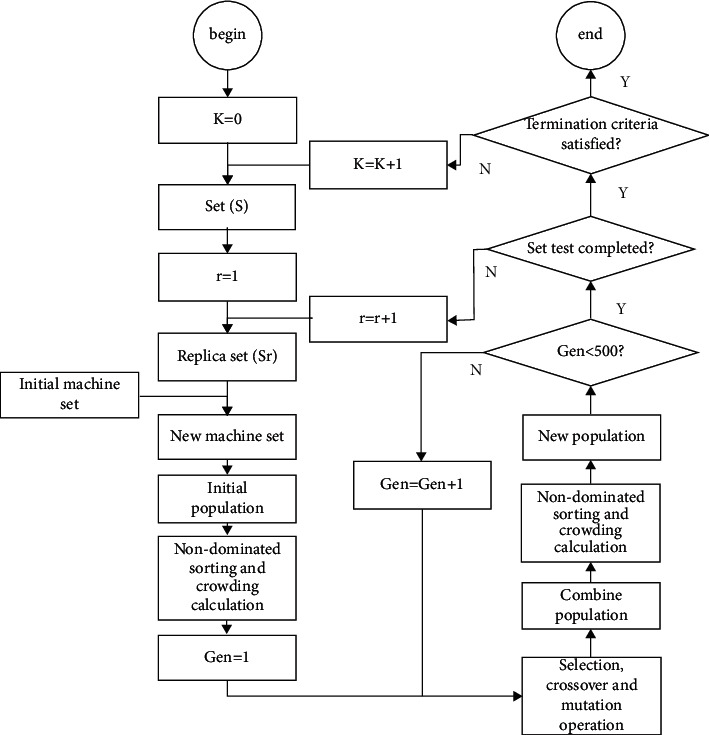
Flowchart for the solution approach.

**Figure 4:**
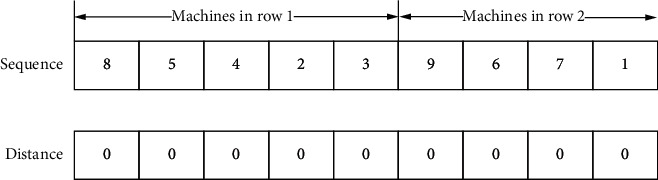
Encoding scheme.

**Figure 5:**
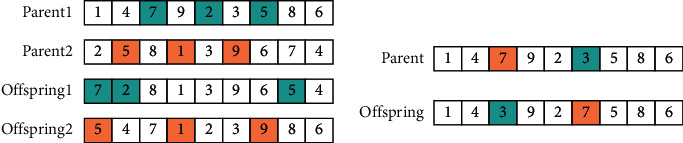
Crossover and mutation of machine sequences.

The solving process of MDRLP-MB is as follows: The first step is to set the increased number K of redundancy, and the value of K increases from 0. In the second step, according to the given K, all possible machine sets are generated by the enumeration method, and according to the method in chapter 3, label one by one, then combine those machine sets and the original machine set, respectively, to generate new machine set. The third step is to initialize the population to obtain the random machine sequence and net distance. According to the machine sequence and net distance, obtain the specific location of the machine and the material flow that minimizes the MHC; then perform nondominated sorting and crowding calculation on it. The fourth step selects, crosses, and mutates the population of the previous step, and the obtained subpopulation merges with the parent population to form a new population; then nondominated sorting and crowding calculation are performed to screen out the new population. The fourth step of the iteration process continues until the stop condition is satisfied. After iterations reach the set value, return to the second step and replace the machine set to generate a new machine set. When all the sets are tested, return to the first step to increase the value of K for calculation until the boost from increasing K becomes too small. The detailed solving process is exhibited in Figure 3:

1. Solution coding: Figure 4 shows the encoding scheme; a layout coding is divided into two sequences. The first sequence represents the arrangement order of all machines; for example, 8 represents the machine number 8. To be more suitable for workshop, production line will wrap automatically when the number of machines reaches or just exceeds half of the total number of machines. If the total number of machines is nine, there will be five machines in the first row and four machines in the second row. The redundancy is also given a number, which is different from the original machine. The second sequence represents the net clearance of the machines (0 represents the distance is 0 meters), where the net clearance plus the minimum distance between two machines is the actual distance. This sequence will be used to fine-tune the distance between the machines so that the machines with large material flows are as close as possible.
2. Population initialization: each sequence in the initial population will be generated separately. Firstly, the first sequence will randomly generate the arrangement order of the machine; secondly, each position of the second sequence will be randomly generated to represent the net distance Δ*k*_*i*_ between adjacent facilities.
3. Genetic operator: the selection method of individuals adopts tournament selection. For the two sequences in the code, different genetic operators are used for crossover and mutation. For machine sequences, Order-Based Crossover (OBX) in Lee and Choi [39] and mutation being the swap mutation are used, as shown in Figure 5. For clearance sequences, arithmetic crossover and real-parameter mutation in [23] are used.
4. Parameter setting: Deb et al. [23] employed 100 populations and 250 generations for NSGA-II. After our test, the optimal set of Pareto is obtained in about 400∼500 generations when the populations are 200. So 500 generations and 200 populations for NSGA-II are employed. The crossover probability uses the same value in [23] and sets the probability as 0.8 to speed up the convergence. Then the termination condition of the entire process is when K equals 4, because when K reaches or exceeds 5, it will take a huge amount of time and is likely to get worse results.

## 5. Experiments

### 5.1. Practical Case Study

We evaluate the performance of the new model by studying a real case. All the data in the case are collected in the factory. The factory has a unit production workshop, it mainly produces auto-related parts and components, and the facility layout is adjusted approximately every month, so the production period is one month (halt of production caused by MB during production is regarded as a failure, for example, a short-term jam of the production machine is not counted as a failure).

In addition, although some of the key machine type in the workshop has redundancy, the factory did not add redundancy to the machine type which has a high probability of failure, resulting in the production halt due to the damage of those types. Inherent availability is obtained by analysis of historical data, as shown in Table 1. The area of the machine is not only the area of the machine itself but the smallest rectangle with a series of areas, including motors, raw material storage areas, and so on, as demonstrated in Table 2.  Table 3 shows the flows between various types in a specific production period. The production halting loss is the same as the calculation of failure; it will be considered as the average sample which is the total loss in several production periods (only the periods when the failure occurred) divided by the number of periods. The loss coefficient D ∗ T of halting production is nearly 1000. The parameter settings are shown in Table 4.

**Figure 6:**
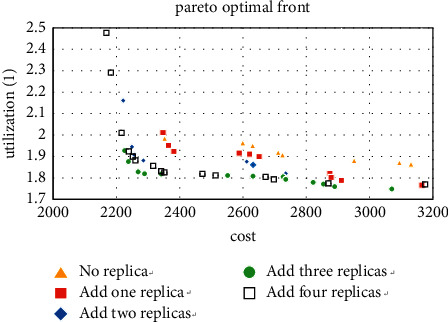
Pareto fronts after adding redundancy.

We use the software MATLAB (version 3.3) to solve this problem and complete the calculation on a computer with Intel(R) Core(TM) i5-4460 CPU @ 3.20 GHz and 8.00 GB. The results obtained by the MATLAB program are shown in Figure 6. The result shows that the Pareto fronts of the layout are optimized after adding redundancy, and when the number of redundancy is 3 and 4, the best curve is obtained. Therefore, decision-makers can add 3 or 4 redundancies to the workshop to get the layout they want.

This paper develops a decision rule to simplify the complexity of the problem. As described in Section 4.1, the more the candidate machine types, the more complicated the problem. In addition, the result above shows that the Pareto fronts are mainly concentrated in partial types. To obtain a relatively reasonable layout, it is necessary to know which machine types should add redundancy (adding redundancy for all machine types is not a reasonable choice).

Use the real-world case above to illustrate the influence of these features in the layout and analyze the factors of each machine type. The factors of the machine type are shown in Table 5.

To find the appropriate machine types for the optimal layout, three features are adopted for analysis.

#### 5.1.1. Looking for Machine Types with a Lower Inherent Availability

The new model points out that there is a differently weighted coefficient as to different breakdown situations. Since the inherent availability of machine types determines the coefficient, the MHC will increase if the machine type has a low inherent availability and it does not have a redundancy. By this way, it is not worth adding redundancy to a machine with a high inherent availability.  Table 6 shows that the inherent availability of the four machine types B, C, D, and E are all greater than the rest of the machines. That is to say, these machine types are more prone to break down than other types. Therefore, consider these 4 machine types as candidates for experimentation.

#### 5.1.2. Evaluating the Material Flow of Each Machine Type

The material flow matrix between each machine is crucial for calculating the MHC in the facility layout. Therefore, evaluating the flow among machines in the layout is reasonable. In addition, it should be noted that each type has multiple redundancies, so the number of connections between different machine types should also be considered. It can be seen in Table 6 that B, C, D, E, and G are relatively more important types; these 5 machine types will be selected first if this factor is considered.

#### 5.1.3. Eliminating Machine Types with a Too Large Area

As we all know, the facility layout should be designed as small as possible. An oversized redundancy will increase the MHC and the area of the workshop, so eliminating E, F, and G whose area is significantly larger than others.

Through analysis, it can be roughly concluded that B, C, and D are unquestionable as alternatives to add redundancy. Comparing the remaining machine types, E and I (with the smallest area) are selected as alternatives. The results show that 74.5% of the Pareto solutions are concentrated in these 5 machine types, and the extreme values of the two optimization objectives are also in these 5 types.

In general, our method is of little significance when the types are small, but for solving large-scale problems, the complexity of the problem leads to extremely low work efficiency. Selecting the best machine types for experiments greatly reduces the complexity of the problem and realizes Pareto solutions with a low inherent availability. Therefore, in the following numerical experiments, for cases with fewer machine types, it could use all machine types to test the effectiveness of this method and use this method to simplify types when there are many machine types.

### 5.2. Numerical Experiments

To check the impact of increasing the redundancy on the layout results by randomly generated cases, to fit the real-world production, the machine types will be based on the previous case in this article, and three numbers {5, 10, 15} will be selected as the machine type. There are three product quantities for each above case, for example, when the machine type is 5, the number of generated products is {2, 4, 6} when the machine type is 10, the number of generated products is {4, 6, 8}, and when the machine type is 15, the number of products is {6, 8, 10}. In this way, numerical experiments include 9 cases for verification, the demand volume of each product is uniformly distributed∼U (100, 300), percentage of passed types for each product is ∼U (0.4, 0.8), each machine type choses ∼U (1, 2) as the length and width, the minimum clearance between the machine obeys the uniform distribution∼U (0.5, 1), and the corridor width is uniformly chosen between 1 and 2. This article believes that the probability of breakdowns and maintenance time are related to the material flows, the greater the material flows passing through the machine type, the greater the probability and maintenance time. Therefore, the generation rule for inherent availability is ∼ U (0.95, 0.99). It is difficult to give loss coefficient a fixed value, because it will vary with the number of products and machines, here the model sets it as *z*_0_/2, where *z*_0_ is the lowest MHC without MB, and Table 6 gives the detailed parameter settings.

**Figure 7:**
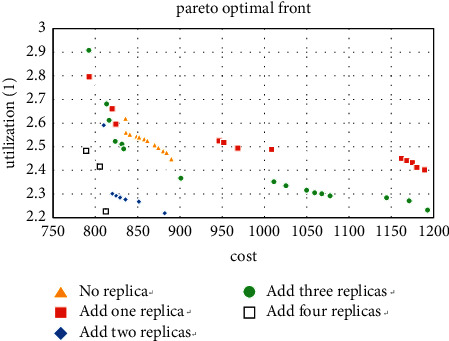
Pareto fronts for set (5 × 2).

**Figure 8:**
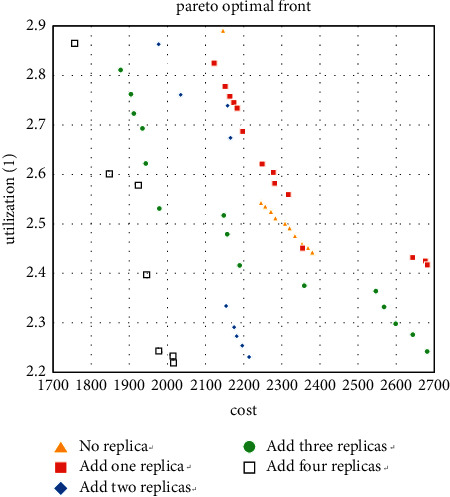
Pareto fronts for set (5 × 4).

**Figure 9:**
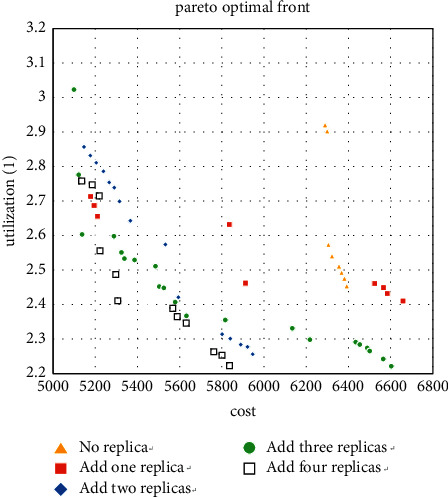
Pareto fronts for set (5 × 6).

We first calculate the case where the machine type is 5 and take all 5 machine types as the types that can add redundancy, and the three Pareto solutions obtained are shown in Figures 7–9.

For the small size problem instances (machine types are 5), it can be seen from Figures 7–9 that the more thevredundancy added, the better the Pareto fronts. On the one hand, it can also be known that the benefit of increasing redundancy is small when there are only two products, and even a worse result is obtained when the number of additions is small. On the other hand, when the number of tasks is 4 and 6 (especially 6), the Pareto fronts are significantly optimized by the number of redundancy. Then use the method in Section 5.1 to analyze the added redundancy and get two candidate machine types. Surprisingly to find these two types occupy 75.6% of the optimal solution, the minimum value of MHC and the maximum utilization value are generated by these two types, respectively. That proves that most of the optimal solutions are concentrated on adding a few types in small-scale cases.

**Figure 10:**
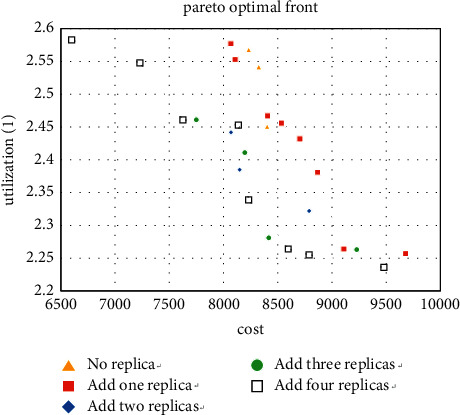
Pareto fronts for set (10 × 4).

**Figure 11:**
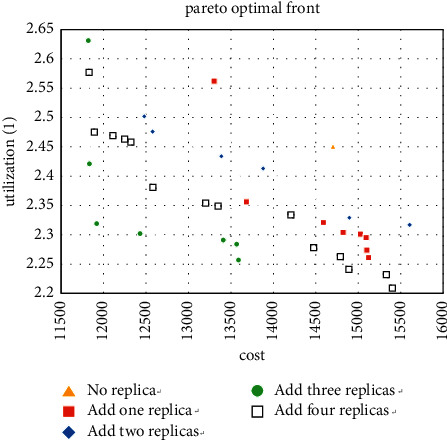
Pareto fronts for set (10 × 6).

**Figure 12:**
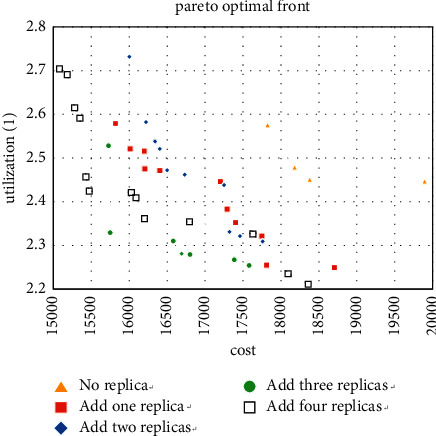
Pareto fronts for set (10 × 8).

For the experiment with the machine type of 10, the improvement of the layout when adding redundancy can be observed from Figures 10–12, especially when the number of copies is 3. The result shows that the five machine types obtained by the method in Section 5.1 account for 71.9% of the Pareto solutions, which also includes the minimum cost and maximum utilization. It further confirms the accuracy of the method.

**Figure 13:**
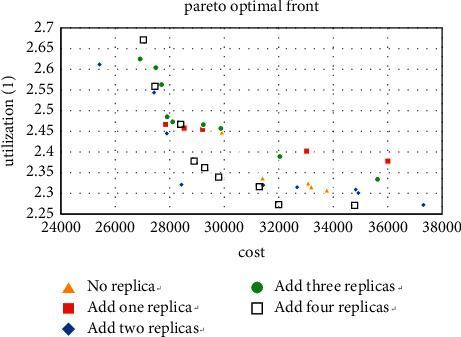
Pareto fronts for set (15 × 6).

**Figure 14:**
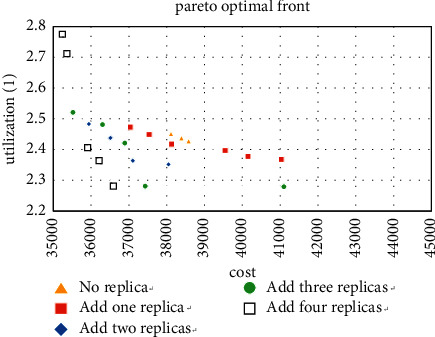
Pareto fronts for set (15 × 8).

**Figure 15:**
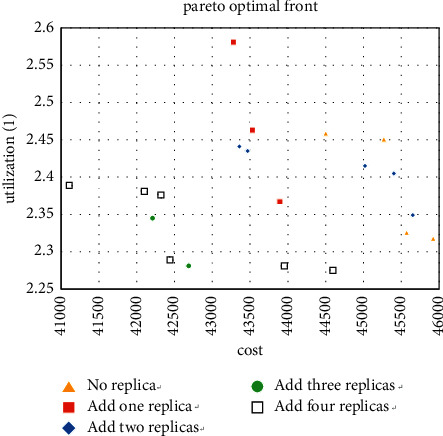
Pareto fronts for set (15 × 10).

The 6 experiments of machine types 5 and 10 support the effectiveness of the decision-making method in Section 5.1. Therefore, when 15 is used as the type number, then use the method in Section 5.1 to obtain the optimal solution while simplifying the calculation, and 5 to 10 types are selected for the experiment. The solutions obtained are shown in Figures 13–15.

For the case where the number of types is 15, it can also be seen that adding redundancy obtains better Pareto solutions. These nine cases show that redundancy significantly reduces the MHC of the layout and increases the workshop utilization to a certain extent, especially when the number of products and machine types is close to each other. Thus, it confirms the effectiveness of the enhanced model.

## 6. Conclusions

This article considers MB which often occurs in production, adding redundancy of some machine type to make the production system more efficient and reliable, and on this basis, a new objective function is proposed to redefine the MHC by optimizing the machine position, the machine gap, and the material flow between machines. After combining the production, a biobjective layout model is organized while minimizing MHC and maximizing the workshop utilization. The increase of candidate machine types in the model will significantly increase the complexity of the problem, so a simple and effective method is proposed to determine the machine type. Numerical results indicate that the preselected type has more than 70% accuracy.

The NSGA-II algorithm is used to solve the MDRLP-MB. The results show that the optimized machine location and machine clearance improve the MHC and the workshop utilization. In addition, this paper also uses a real-world production case to explain the model's parameters with factory production conditions, and the example shows that the result is satisfactory. The cases that were randomly generated further show that the enhanced model also effectively solves different scale problems. In conclusion, this model can solve the layout problem better for enterprises with frequent failures or huge shutdown losses. Finally, future research can turn to the layout problem of more rows or more units and handle different fault situations differently.

## Figures and Tables

**Table 1:** The inherent availability of machine types.

Facility type	A	B	C	D	E	F	G	H	I	J
Inherent availability (*A*_r_)	0.97	0.955	0.96	0.95	0.96	0.97	0.97	0.99	0.97	0.98

**Table 2:** Dimensions of machine types (meter).

Facility type	A	B	C	D	E	F	G	H	I	J
Length (*l*_i_)	5.2	4.2	2.0	3.6	5.3	4.5	4.0	3.0	2.2	4.3
Width (*w*_*i*_)	2.1	2.6	5.1	2.6	3.5	3.5	5.0	2.8	3.2	2.7

**Table 3:** Material flows between machine types.

Facility type	A	B	C	D	E	F	G	H	I	J
A	0	30	10	40	0	0	0	0	0	0
B	30	0	55	15	35	25	0	0	0	0
C	10	55	0	45	0	0	0	0	0	0
D	40	15	30	0	65	20	15	0	0	0
E	0	35	45	65	0	30	10	15	25	0
F	0	25	0	20	30	0	30	0	0	0
G	0	0	0	15	10	30	0	15	55	0
H	0	0	0	0	15	0	15	0	15	15
I	0	0	0	0	25	0	55	15	0	0
J	0	0	0	0	0	0	0	15	0	0

**Table 4:** Other parameters.

Corridor width (C)	1

Clearance (Δ*l*_*ij*_)	0.5
Loss coefficient (*D ∗ T*)	1000

**Table 5:** The factors table of machine types.

Facility type	A	B	C	D	E	F	G	H	I	J
Material flow	80 (8)	160 (3)	140 (4)	200 (1)	180 (2)	105 (6)	125 (5)	60 (9)	95 (7)	15 (10)
Connections	3 (8)	5 (3)	4 (5)	6 (1)	6 (1)	4 (5)	5 (3)	4 (5)	3 (8)	1 (10)
Inherent availability	0.97 (5)	0.955 (2)	0.96 (3)	0.95 (1)	0.96 (3)	0.97 (5)	0.97 (5)	0.99 (10)	0.97 (5)	0.98 (9)
Area	10.92 (5)	10.92 (5)	10.2 (4)	9.36 (3)	18.55 (9)	15.75 (8)	20 (10)	8.4 (2)	7.04 (1)	11.61 (7)

**Table 6:** Parameter settings for generating test problem instances.

Machine types	5	10	15
Product types	{2, 4, 6}	{4, 6, 8}	{6, 8, 1 0}
Demand	∼U (100, 300)		
Machine width	∼U (1, 2)		
Machine depth			
Corridor width	∼U (1, 2)		
Clearance	∼U (0.5, 1)		
Process flow length	∼U (0.4, 0.8)		
Inherent availability	∼U (0.95, 0.99)		
Loss coefficient (D ∗ T)	*z*_0_/2		

## Data Availability

The datasets used during the current study are available from the corresponding author on reasonable request. The code used during the current study is available from the corresponding author on reasonable request.

## Supplementary Materials

Supplementary Materials The supplementary materials contain the function of nsga_2_optimization.
