# Supplementary material for: An Enhanced Multiobjective Double Row Layout Model considering the Machine Breakdowns
Source: Comput Intell Neurosci. 2022 Aug 28;2022:6289609. doi: 10.1155/2022/6289609 (PMC9441348; doi:10.1155/2022/6289609)
Supplement: Supplementary Materials — The supplementary materials contain the function of nsga_2_optimization. [file 6289609.f1.docx]

function nsga_2_optimization

pop = 200; %种群数量

gen =500;

%迭代次数

M = 2; %目标函数数量

v=[1 2 3 4 5];%备选型号

% v=[1 2 3 4 5 6 7 8 9 10];%备选型号

v0=5;

s=cell(1);

for i=0:4

% i=0;

t1=clock;

V=5+i;

if i~=0

s=fun(i,v0);

t0=length(s);

else

t0=1;

end

bestfit=10000;

bestdis=1000;

bestrand1=[];

bestrand2=[];

chromosome1=[];

chromosome2=[];

parfor j=1:t0

% 实际案例

% p=[0.03 0.045 0.04 0.05 0.04 0.03 0.03 0.01 0.03 0.02];

% w=[5.2 4.2 2.0 3.6 5.3 4.5 4.0 3.0 2.2 4.3];

% h=[2.1 2.6 5.1 2.6 3.5 3.5 5.0 2.8 3.2 2.7];

% 案例1

% p=[0.04 0.02 0.05 0.02 0.03];

% w=[1.5 2 2 1.5 1 ];

% h=[1.5 2 2 1.5 1 ];

% 案例2

% p=[0.04 0.01 0.04 0.02 0.03];

% w=[1.5 2 2 1.5 1 ];

% h=[1.5 2 2 1.5 1 ];

% 案例3

p=[0.03 0.01 0.04 0.02 0.02];

w=[1.5 2 2 1.5 1 ];

h=[1.5 2 2 1.5 1 ];

% 案例4

% p=[0.2 0.15 0.2 0.15 0.15 0.05 0.1 0.1 0.15 0.05 ];

% w=[1.5 2 2 1.5 1 1.5 2 2 1.5 1 ];

% h=[1.5 2 2 1.5 1 1.5 2 2 1.5 1 ];

% 案例5

% p=[0.2 0.15 0.2 0.15 0.15 0.05 0.1 0.05 0.15 0.05];

% w=[1.5 2 2 1.5 1 1.5 2 2 1.5 1 ];

% h=[1.5 2 2 1.5 1 1.5 2 2 1.5 1 ];

% 案例6

% p=[0.2 0.15 0.2 0.15 0.15 0.05 0.1 0.05 0.15 0.05];

% w=[1.5 2 2 1.5 1 1.5 2 2 1.5 1 ];

% h=[1.5 2 2 1.5 1 1.5 2 2 1.5 1 ];

%案例8

% p=[0.15 0.2 0.2 0.15 0.2 0.1 0.2 0.15 0.1 0.15 0.15 0.05 0.1 0.1 0.15];

% w=[1.5 2 2 1.5 1 1.5 2 2 1.5 1 1 1.5 2 2 1.5 ];

% h=[1.5 2 2 1.5 1 1.5 2 2 1.5 1 1 1.5 2 2 1.5 ];

%案例9

% p=[0.15 0.2 0.2 0.15 0.2 0.1 0.2 0.15 0.1 0.15 0.15 0.05 0.05 0.1 0.15];

% w=[1.5 2 2 1.5 1 1.5 2 2 1.5 1 1 1.5 2 2 1.5 ];

% h=[1.5 2 2 1.5 1 1.5 2 2 1.5 1 1 1.5 2 2 1.5 ];

if i~=0

a=0;

r=cell(1,length(v));

for k=1:length(s{j})

r{k}(1,1)=v(k);

if s{j}(1,k)~=0

for m=1:s{j}(1,k)

a=a+1;

r{k}(1,m+1)=V-i+a;

w(V-i+a)=w(v(k));

h(V-i+a)=h(v(k));

p(V-i+a)=p(v(k));

end

end

end

k0=0;

else

r=zeros(1,5);

k0=1;

end

chromosome = initialize_variables(pop, M, V, w, h, p, r, k0);%初始化种群

chromosome = non_domination_sort_mod(chromosome, M, V);%初始化种群非支配快速排序和拥挤度计算

for ii = 1 : gen

pool = round(pop/2);%round() 四舍五入取整 交配池大小

tour = 2;%竞标赛 参赛选手个数

parent_chromosome = tournament_selection(chromosome, pool, tour);%竞标赛选择适合繁殖的父代

offspring_chromosome1 = mutation(parent_chromosome,M, V, w, h, p, r, k0);

offspring_chromosome =crossover(offspring_chromosome1,M, V, w, h, p, r, k0);

[main_pop,~] = size(chromosome);%父代种群的大小

[offspring_pop,~] = size(offspring_chromosome);%子代种群的大小

intermediate_chromosome=zeros(main_pop+offspring_pop,M+2*V);

intermediate_chromosome(1:main_pop,1:M+2*V) = chromosome(:,1:M+2*V);

intermediate_chromosome(main_pop + 1 : main_pop + offspring_pop,1:M+2*V) = offspring_chromosome(:,1:M+2*V);%合并种群

intermediate_chromosome = non_domination_sort_mod(intermediate_chromosome, M, V);%快速非支配排序

chromosome = replace_chromosome(intermediate_chromosome, M, V, pop);%选择N个

end

[px,~]=size(chromosome);

if i~=0

b=s{j};

chromosome0=[];

for pp=1:px

chromosome0(pp,:) =[chromosome(pp,:),b];

end

else

chromosome0=chromosome;

end

chromosome1=[chromosome0;chromosome1];

end

chromosome1 = non_domination_sort_mod(chromosome1, M, V);

[px,~]=size(chromosome1);

dd=0;

for u=1:px

if chromosome1(u,2*V + 3)==1

dd=dd+1;

chromosome2(dd,:)=chromosome1(u,:);

end

end

[px,~]=size(chromosome2);

bestfit=min(chromosome2(:,2*V+1));

bestdis=min(chromosome2(:,2*V+2));

switch i

case 0

chromosome00=chromosome2;

plot(chromosome2(:,2*V + 1),chromosome2(:,2*V + 2),'square');

case 1

chromosome01=chromosome2;

plot(chromosome2(:,2*V + 1),chromosome2(:,2*V + 2),'pentagram');

case 2

chromosome02=chromosome2;

plot(chromosome2(:,2*V + 1),chromosome2(:,2*V + 2),'x');

case 3

chromosome03=chromosome2;

plot(chromosome2(:,2*V + 1),chromosome2(:,2*V + 2),'o');

otherwise

chromosome04=chromosome2;

plot(chromosome2(:,2*V + 1),chromosome2(:,2*V + 2),'+');

end

xlabel('Cost'); ylabel('1/Utilization');

title('Pareto Optimal Front');

hold on;

disp(num2str(etime(clock,t1)));

disp(bestfit);

disp(bestdis);

disp(bestrand1);

disp(bestrand2);

t2=clock;

etime(t2,t1);

end

legend('no replica','add one replica','add two replicas','add three replicas','add four replicas');

end

function s=fun(i,v0)

% i=3;

% v0=5;

for j=1:v0

aa(j,:)=randperm(i);

aaa{j}=[aa(j,:),0];

end

nx=v0;

ny=i;

xxx=1 ; %行数

yyy=[];

global nnn; %组合序号

nnn=1;

global zuhe; %存放所有组合

zuhe={};

for j=aaa{xxx}

yyy(xxx)=j ;

diedai( aaa, xxx, nx, yyy);

end

k=1;

for j=1:length(zuhe)

if sum(zuhe{j})==i

s{k}=zuhe{j};

k=k+1;

end

end

end

function f= evaluate_objective(x, M, V, w, h, p, r, k0)

% w=[3.0 4.0 3.0 6.0 4.0 6.5 4.0 3.0 4.2 4.0 9.0 4.0 5.0 4.0 3.0];

% h=[3.0 4.0 3.0 6.0 4.0 6.5 4.0 3.0 4.2 4.0 9.0 4.0 5.0 4.0 3.0];

% x=[1 3 4 8 9 12 6 10 5 2 7 13 11 15 14 0 0 0 0 0 0 0 0 0 0 0 0 0 0 0];

% p=[0.03 0.045 0.04 0.05 0.04 0.03 0.03 0.01 0.03 0.02 0.03 0.03 0.01 0.03 0.02];

% M=2;

% V=15;

f=zeros(1,M);

point=round(V/2);

layout{1}=x(1:point);

layout{2}=x(point+1:V);

o=zeros(1,V);

t0=zeros(1,V);

Y=zeros(1,2);

D=zeros(V);

X=0;

for nr=1:2

o(layout{nr}(1))=0.5+w(layout{nr}(1))/2+x(layout{nr}(1)+V);

t0(layout{nr}(1))=nr;

Y(nr)=h(layout{nr}(1));

for nc=2:length(layout{nr})

o(layout{nr}(nc))=o(layout{nr}(nc-1))+(w(layout{nr}(nc-1))+w(layout{nr}(nc)))/2+x(layout{nr}(nc)+V)+0.5;

t0(layout{nr}(nc))=nr;

if X<o(layout{nr}(nc))+w(layout{nr}(nc))/2

X=o(layout{nr}(nc))+w(layout{nr}(nc))/2;

end

if Y(nr)<h(layout{nr}(nc))

Y(nr)=h(layout{nr}(nc));

end

end

end

T=0;

for uu=1:V

T=w(uu)*h(uu)+T;

end

f(2)=((Y(1)+Y(2)+2)*(X+0.5))/T;

for ii=1:V

for jj=1:V

if t0(ii)~=t0(ii)

D(jj,ii)=abs((o(ii)-o(jj)))+2;

else

D(jj,ii)=abs((o(ii)-o(jj)));

end

end

end

H=cell(1,2);

H{1}=zeros(V);

H{2}=zeros(V);

H{3}=zeros(V);

H{4}=zeros(V);

H{5}=zeros(V);

H{6}=zeros(V);

% H{7}=zeros(V);

% H{8}=zeros(V);

% H{9}=zeros(V);

% H{10}=zeros(V);

% 实际案例

% [H{1}(1,2),H{1}(2,3),H{1}(3,4),H{1}(4,5),H{1}(5,6),H{1}(6,7),H{1}(7,9)]=deal(30);

% [H{2}(2,3),H{2}(3,4),H{2}(4,5),H{2}(5,8),H{2}(8,9)]=deal(15);

% [H{3}(2,4),H{3}(4,7),H{3}(7,8),H{3}(8,10)]=deal(15);

% [H{4}(1,3),H{4}(3,2),H{4}(2,5),H{4}(5,7)]=deal(10);

% [H{5}(6,4),H{5}(4,1),H{5}(1,4),H{5}(4,5)]=deal(20);

% [H{6}(7,9),H{6}(9,5),H{6}(5,2),H{6}(2,6)]=deal(25);

% 案例1

% [H{1}(1,3),H{1}(3,5)]=deal(300);

% [H{2}(3,1),H{2}(1,4),H{2}(4,2)]=deal(100);

% 案例2

% [H{1}(3,1),H{1}(1,5)]=deal(300);

% [H{2}(4,2),H{2}(2,1)]=deal(200);

% [H{3}(5,3),H{3}(3,2)]=deal(200);

% [H{4}(1,3),H{4}(3,4),H{4}(4,2)]=deal(100);

%案例3

[H{1}(4,2),H{1}(2,3)]=deal(200);

[H{2}(5,2),H{2}(2,1)]=deal(300);

[H{3}(5,4),H{3}(4,3)]=deal(100);

[H{4}(3,1),H{4}(1,4),H{4}(4,5)]=deal(300);

[H{5}(4,1),H{5}(1,5),H{5}(5,2)]=deal(200);

[H{6}(4,3),H{6}(3,1),H{6}(1,2)]=deal(300);

% 案例4

% [H{1}(3,1),H{1}(1,5),H{1}(5,2)]=deal(200);

% [H{2}(10,9),H{2}(9,4),H{2}(4,3),H{2}(3,1),H{2}(1,7)]=deal(200);

% [H{3}(9,2),H{3}(2,1),H{3}(1,8),H{3}(8,3),H{3}(3,5),H{3}(5,4)]=deal(300);

% [H{4}(8,3),H{4}(3,1),H{4}(1,7),H{4}(7,4),H{4}(4,2),H{4}(2,6),H{4}(6,5)]=deal(100);

% 案例5

% [H{1}(3,1),H{1}(1,5),H{1}(5,2)]=deal(200);

% [H{2}(10,9),H{2}(9,4),H{2}(4,3),H{2}(3,1),H{2}(1,7)]=deal(200);

% [H{3}(9,2),H{3}(2,1),H{3}(1,8),H{3}(8,3),H{3}(3,5),H{3}(5,4)]=deal(300);

% [H{4}(8,3),H{4}(3,1),H{4}(1,7),H{4}(7,4),H{4}(4,2),H{4}(2,6),H{4}(6,5)]=deal(100);

% [H{5}(6,9),H{5}(9,1),H{5}(1,2),H{5}(2,5)]=deal(300);

% [H{6}(4,1),H{6}(1,3),H{6}(3,9),H{6}(9,7)]=deal(200);

% 案例6

% [H{1}(3,1),H{1}(1,5),H{1}(5,2)]=deal(200);

% [H{2}(10,9),H{2}(9,4),H{2}(4,3),H{2}(3,1),H{2}(1,7)]=deal(200);

% [H{3}(9,2),H{3}(2,1),H{3}(1,8),H{3}(8,3),H{3}(3,5),H{3}(5,4)]=deal(300);

% [H{4}(8,3),H{4}(3,1),H{4}(1,7),H{4}(7,4),H{4}(4,2),H{4}(2,6),H{4}(6,5)]=deal(100);

% [H{5}(6,9),H{5}(9,1),H{5}(1,2),H{5}(2,5)]=deal(300);

% [H{6}(4,1),H{6}(1,3),H{6}(3,9),H{6}(9,7)]=deal(200);

% [H{7}(5,2),H{7}(2,1),H{7}(1,3),H{7}(3,4)]=deal(200);

% [H{8}(6,7),H{8}(7,8),H{8}(8,1),H{8}(1,2)]=deal(100);

% 案例8

% [H{1}(5,7),H{1}(7,8),H{1}(8,11),H{1}(11,15),H{1}(15,14)]=deal(300);

% [H{2}(1,2),H{2}(2,4),H{2}(4,9),H{2}(9,12),H{2}(12,15),H{2}(15,13),H{2}(13,3),H{2}(3,5)]=deal(200);

% [H{3}(3,7),H{3}(7,2),H{3}(2,11),H{3}(11,10),H{3}(10,6),H{3}(6,1),H{3}(1,4),H{3}(4,5)]=deal(200);

% [H{4}(7,9),H{4}(9,8),H{4}(8,1),H{4}(1,3),H{4}(3,4),H{4}(4,2),H{4}(2,10),H{4}(10,11),H{4}(11,15)]=deal(100);

% [H{5}(4,3),H{5}(3,2),H{5}(2,1),H{5}(1,8),H{5}(8,5),H{5}(5,10),H{5}(10,13),H{5}(13,14),H{5}(14,11)]=deal(200);

% [H{6}(11,13),H{6}(13,1),H{6}(1,7),H{6}(7,4),H{6}(4,3),H{6}(3,8),H{6}(8,5),H{6}(5,2),H{6}(2,14),H{6}(14,10)]=deal(100);

% [H{7}(3,2),H{7}(2,5),H{7}(5,8),H{7}(8,10),H{7}(10,11),H{7}(11,7),H{7}(7,6)]=deal(200);

% [H{8}(2,3),H{8}(3,6),H{8}(6,7),H{8}(7,9),H{8}(9,12),H{8}(12,15),H{8}(15,10)]=deal(200);

% 案例8

% [H{1}(5,7),H{1}(7,8),H{1}(8,11),H{1}(11,15),H{1}(15,14)]=deal(300);

% [H{2}(1,2),H{2}(2,4),H{2}(4,9),H{2}(9,12),H{2}(12,15),H{2}(15,13),H{2}(13,3),H{2}(3,5)]=deal(200);

% [H{3}(3,7),H{3}(7,2),H{3}(2,11),H{3}(11,10),H{3}(10,6),H{3}(6,1),H{3}(1,4),H{3}(4,5)]=deal(200);

% [H{4}(7,9),H{4}(9,8),H{4}(8,1),H{4}(1,3),H{4}(3,4),H{4}(4,2),H{4}(2,10),H{4}(10,11),H{4}(11,15)]=deal(100);

% [H{5}(4,3),H{5}(3,2),H{5}(2,1),H{5}(1,8),H{5}(8,5),H{5}(5,10),H{5}(10,13),H{5}(13,14),H{5}(14,11)]=deal(200);

% [H{6}(11,13),H{6}(13,1),H{6}(1,7),H{6}(7,4),H{6}(4,3),H{6}(3,8),H{6}(8,5),H{6}(5,2),H{6}(2,14),H{6}(14,10)]=deal(100);

% [H{7}(3,2),H{7}(2,5),H{7}(5,8),H{7}(8,10),H{7}(10,11),H{7}(11,7),H{7}(7,6)]=deal(200);

% [H{8}(2,3),H{8}(3,6),H{8}(6,7),H{8}(7,9),H{8}(9,12),H{8}(12,15),H{8}(15,10)]=deal(200);

% [H{9}(5,7),H{9}(7,12),H{9}(12,15),H{9}(15,11),H{9}(11,4),H{9}(4,2),H{9}(4,9)]=deal(100);

% [H{10}(8,1),H{10}(1,5),H{10}(5,9),H{10}(9,4),H{10}(4,6),H{10}(6,2),H{10}(2,7),H{10}(7,10),H{10}(10,14)]=deal(100);

t=1;

for i=1:V

t=t*(1-p(i));

end

% k0=0;

% r{1}=[1 10];

% r{2}=[4];

% r{3}=[6];

% r{4}=[8];

if k0==0

yp=length(r);

for i=1:yp

C{i}=zeros(1,length(r{i}));

if length(r{i})>1

for j=1:(length(r{i})-1)

k=[nchoosek(r{i},j),zeros(length(nchoosek(r{i},j)),length(r{i})-j)];

C{i}= [C{i};k];

end

end

end

nx=length(r);

xxx=1 ; %行数

yyy={};

global nnn; %组合序号

nnn=1;

global zuhe; %存放所有组合

zuhe={};

for j=1:length(C{xxx})

yyy{xxx}=C{xxx}(j,:);

diedai0( C, xxx, nx, yyy);

end

y=zuhe;

tp=length(y);

g=zeros(1,tp);

P=zeros(1,tp);

pp=zeros(1,tp);

for i=1:tp

a=0;

t0=1;

xx=zeros(1,20);

for j=1:length(y{i})

if y{i}(1,j)~=0

a=a+1;

xx(a)=y{i}(1,j);

t0=t0*p(xx(a))/(1-p(xx(a)));

end

end

g0=newH(xx,D,r,H);

g(i)=g0;

P(i)=t*t0;

end

q=sum(P);

f(1)=(1-q)*3/2*g(1);

for j=1:length(P)

f(1) = f(1)+P(j)*g(j);

end

else

f(1)=t*(sum(sum(H{1}.*D+H{2}.*D+H{3}.*D+H{4}.*D+H{5}.*D+H{6}.*D)))+(1-t)*3/2*(sum(sum(H{1}.*D+H{2}.*D+H{3}.*D+H{4}.*D+H{5}.*D+H{6}.*D)));

% f(1)=t*(sum(sum(H{1}.*D+H{2}.*D)))+(1-t)*11/10*(sum(sum(H{1}.*D+H{2}.*D)));

% f(1)=t*(sum(sum(H{1}.*D+H{2}.*D+H{3}.*D+H{4}.*D)))+(1-t)*11/10*(sum(sum(H{1}.*D+H{2}.*D+H{3}.*D+H{4}.*D)));

% f(1)=t*(sum(sum(H{1}.*D+H{2}.*D+H{3}.*D+H{4}.*D+H{5}.*D+H{6}.*D+H{7}.*D+H{8}.*D)))+(1-t)*11/10*(sum(sum(H{1}.*D+H{2}.*D+H{3}.*D+H{4}.*D+H{5}.*D+H{6}.*D+H{7}.*D+H{8}.*D)));

% f(1)=t*(sum(sum(H{1}.*D+H{2}.*D+H{3}.*D+H{4}.*D+H{5}.*D+H{6}.*D+H{7}.*D+H{8}.*D+H{9}.*D+H{10}.*D)))+(1-t)*11/10*(sum(sum(H{1}.*D+H{2}.*D+H{3}.*D+H{4}.*D+H{5}.*D+H{6}.*D+H{7}.*D+H{8}.*D+H{9}.*D+H{10}.*D)));

end

end

function offspring_chromosome =crossover(offspring_chromosome1,M, V, w, h, p, r, k0)

[px,~]=size(offspring_chromosome1);

% V=9;

% for i=1:50

% offspring_chromosome1(i,1:V)=randperm(V);

% for j=V:2*V

% offspring_chromosome1(i,j)=rand(1);

% end

% end

% px=50;

offspring_chromosome=offspring_chromosome1;

% c1=zeros(1,V);

% c2=zeros(1,V);

for i=1:2:(px-1)

% i=6;

if rand(1)<0.9

a1=randperm(V);

a2=randperm(V);

b1=a1(1:3);

b2=a2(1:3);

k1=0;

for j1=1:V

if ismember(offspring_chromosome1(i+1,j1),b1)

continue;

else

k1=k1+1;

c1(k1)=offspring_chromosome1(i+1,j1);

end

end

k=0;

for j3=1:V

if ismember(offspring_chromosome1(i,j3),b1)

offspring_chromosome(i,j3)=offspring_chromosome1(i,j3);

else

k=k+1;

offspring_chromosome(i,j3)=c1(k);

end

end

k2=0;

for j2=1:V

if ismember(offspring_chromosome1(i,j2),b2)

continue;

else

k2=k2+1;

c2(k2)=offspring_chromosome1(i,j2);

end

end

k=0;

for j4=1:V

if ismember(offspring_chromosome1(i+1,j4),b2)

offspring_chromosome(i+1,j4)=offspring_chromosome1(i+1,j4);

else

k=k+1;

offspring_chromosome(i+1,j4)=c2(k);

end

end

t=unidrnd(2,1,V)-1;

c1=offspring_chromosome1(i,V+1:2*V);

c2=offspring_chromosome1(i+1,V+1:2*V);

offspring_chromosome(i,V+1:2*V)=c1.*t+c2.*(1-t);

offspring_chromosome(i+1,V+1:2*V)=c1.*(1-t)+c2.*t;

offspring_chromosome(i,2*V + 1: M + 2*V) = evaluate_objective(offspring_chromosome(i,:), M, V, w, h, p, r, k0);

offspring_chromosome(i+1,2*V + 1: M + 2*V) = evaluate_objective(offspring_chromosome(i,:), M, V, w, h, p, r, k0);

end

end

end

function []=diedai(aaa,xxx,nx,yyy)

global zuhe

global nnn

xxx=xxx+1;

for i=aaa{xxx}

yyy(xxx)=i;

if xxx<nx

diedai(aaa,xxx,nx,yyy);

end

if xxx==nx

zuhe{nnn}=yyy;

nnn=nnn+1;

end

end

end

function []=diedai0(C,xxx,nx,yyy)

global zuhe

global nnn

xxx=xxx+1;

for i=1:length(C{xxx})

yyy{xxx}=C{xxx}(i,:);

if xxx<nx

diedai0(C,xxx,nx,yyy);

end

if xxx==nx

zuhe{nnn}=zeros(1,0);

for k=1:nx

zuhe{nnn}=[zuhe{nnn},yyy{k}];

end

nnn=nnn+1;

end

end

end

function []=diedai1(r,xxx,nx,xx,i0,D,yyy,H)

global a

global bestH

xxx=xxx+1;

for i=1:length(r{xxx})

if ismember(r{xxx}(1,i),xx)

continue;

end

yyy(xxx)=i;

if xxx<nx

diedai1(r,xxx,nx,xx,i0,D,yyy,H);

end

if xxx==nx

H0{i0}=H{i0};

for k=1:nx

H0{i0}(r{k}(1,yyy(k)),:)=H0{i0}(r{k}(1,1),:);

H0{i0}(:,r{k}(1,yyy(k)))=H0{i0}(:,r{k}(1,1));

if yyy(k)~=1

H0{i0}(r{k}(1,1),:)=0;

H0{i0}(:,r{k}(1,1))=0;

end

end

if a(i0)>sum(sum(D.*H0{i0}))

a(i0)=sum(sum(D.*H0{i0}));

bestH{i0}=H0{i0};

end

end

end

end

function f = initialize_variables(N, M, V, w, h, p, r, k0)

% M=2;

% N=10;

% V=10;

K = M + 2*V;

f=zeros(N,K);

for i = 1 : N

f(i,1:V)=randperm(V);

for j=V+1:2*V

f(i,j)=0;

end

f(i,(2*V + 1): K) = evaluate_objective(f(i,:), M, V, w, h, p, r, k0);

end

end

function f = mutation(parent_chromosome, M, V, w, h, p, r,k0)

% M=2;

% N=10;

% V=11;

% K = M + 2*V;

% w=[3.0 4.0 3.0 6.0 4.0 6.5 4.0 3.0 4.2 4.0 9.0];

% h=[3.0 4.0 3.0 6.0 4.0 6.5 4.0 3.0 4.2 4.0 9.0];

% x=[1 3 4 8 9 10 6 11 5 2 7 0 0 0 0 0 0 0 0 0 0 0];

% p=[0.1 0.1 0.1 0.1 0.2 0.2 0.2 0.2 0.2 0.2 0.2];

% r{1}=[1 10];

% r{2}=[5 11];

% r{3}=[9];

% for i = 1 : N

% f(i,1:V)=randperm(V);

% for j=V+1:2*V

% f(i,j)=0;

% end

% end

% parent_chromosome=f;

[px,~] = size(parent_chromosome);

child=parent_chromosome;

for i = 1:px

if(rand<0.5)

j=unidrnd(V);

k=unidrnd(V);

child(i,:)=parent_chromosome(i,:);

child(i,j)=parent_chromosome(i,k);

child(i,k)=parent_chromosome(i,j);

else

t=randperm(V);

k00=unidrnd(V);

for j00=1:k00

child(i,t(j00)+V)=unidrnd(100,1)/100;

end

end

child(i,2*V + 1: M + 2*V) = evaluate_objective(child(i,:), M, V, w, h, p, r, k0);

end

f = child;

end

function g0=newH(xx,D,r,H)

% xx=[10 12 14];

% for i=1:14

% for j=1:14

% D(i,j)=i+j;

% end

% end

% V=14;

% r{1}=[1 10];

% r{2}=[4 ];

% r{3}=[7 11 12];

% r{4}=[9 13 14];

% H=cell(1,6);

% H{1}=zeros(V);

% H{2}=zeros(V);

% H{3}=zeros(V);

% H{4}=zeros(V);

% H{5}=zeros(V);

% H{6}=zeros(V);

% [H{1}(1,2),H{1}(2,3),H{1}(3,4),H{1}(4,5),H{1}(5,6),H{1}(6,7),H{1}(7,9)]=deal(30);

% [H{2}(2,3),H{2}(3,4),H{2}(4,5),H{2}(5,8),H{2}(8,9)]=deal(15);

% [H{3}(2,4),H{3}(4,7),H{3}(7,8),H{3}(8,10)]=deal(15);

% [H{4}(1,3),H{4}(3,2),H{4}(2,5),H{4}(5,7)]=deal(10);

% [H{5}(6,4),H{5}(4,1),H{5}(1,4),H{5}(4,5)]=deal(20);

% [H{6}(7,9),H{6}(9,5),H{6}(5,2),H{6}(2,6)]=deal(25);

global a;

global bestH;

bestH={};

% a=[100000,100000,100000,100000];

a=[100000,100000,100000,100000,100000,100000];

% a=[100000,100000];

% a=[100000,100000,100000,100000,100000,100000,100000,100000];

% a=[100000,100000,100000,100000,100000,100000,100000,100000,100000,100000];

nx=length(r);

% for i0=1:2

for i0=1:6

yyy=[];

xxx=1;

for i=1:length(r{xxx})

if ismember(r{xxx}(1,i),xx)

continue;

end

yyy(xxx)=i;

diedai1(r,xxx,nx,xx,i0,D,yyy,H);

end

end

% g0=a(1)+a(2)+a(3)+a(4);

g0=a(1)+a(2)+a(3)+a(4)+a(5)+a(6);

% g0=a(1)+a(2)+a(3)+a(4)+a(5)+a(6)+a(7)+a(8);

% g0=a(1)+a(2)+a(3)+a(4)+a(5)+a(6)+a(7)+a(8)+a(9)+a(10);

% % g0=a(1)+a(2);

end

%% 对初始种群开始排序 快速非支配排序

% 使用非支配排序对种群进行排序。该函数返回每个个体对应的排序值和拥挤距离，是一个两列的矩阵。

% 并将排序值和拥挤距离添加到染色体矩阵中

function f = non_domination_sort_mod(x, M, V)

[N, ~] = size(x);% N为矩阵x的行数，也是种群的数量

clear m

front = 1;

F(front).f = [];

individual = [];

V=2*V;

for i = 1 : N

individual(i).n = 0;%n是个体i被支配的个体数量

individual(i).p = [];%p是被个体i支配的个体集合

for j = 1 : N

dom_less = 0;

dom_equal = 0;

dom_more = 0;

for k = 1 : M %判断个体i和个体j的支配关系

if (x(i,V + k) < x(j,V + k))

dom_less = dom_less + 1;

elseif (x(i,V + k) == x(j,V + k))

dom_equal = dom_equal + 1;

else

dom_more = dom_more + 1;

end

end

if dom_less == 0 && dom_equal ~= M % 说明i受j支配，相应的n加1

individual(i).n = individual(i).n + 1;

elseif dom_more == 0 && dom_equal ~= M % 说明i支配j,把j加入i的支配合集中

individual(i).p = [individual(i).p j];

end

end

if individual(i).n == 0 %个体i非支配等级排序最高，属于当前最优解集，相应的染色体中携带代表排序数的信息

x(i,M + V + 1) = 1;

F(front).f = [F(front).f i];%等级为1的非支配解集

end

end

%上面的代码是为了找出等级最高的非支配解集

%下面的代码是为了给其他个体进行分级

while ~isempty(F(front).f)

Q = []; %存放下一个front集合

for i = 1 : length(F(front).f)%循环当前非支配解集中的个体

if ~isempty(individual(F(front).f(i)).p)%个体i有自己所支配的解集

for j = 1 : length(individual(F(front).f(i)).p)%循环个体i所支配解集中的个体

individual(individual(F(front).f(i)).p(j)).n = ...%...表示的是与下一行代码是相连的， 这里表示个体j的被支配个数减1

individual(individual(F(front).f(i)).p(j)).n - 1;

if individual(individual(F(front).f(i)).p(j)).n == 0% 如果q是非支配解集，则放入集合Q中

x(individual(F(front).f(i)).p(j),M + V + 1) = ...%个体染色体中加入分级信息

front + 1;

Q = [Q individual(F(front).f(i)).p(j)];

end

end

end

end

front = front + 1;

F(front).f = Q;

end

[temp,index_of_fronts] = sort(x(:,M + V + 1));%对个体的代表排序等级的列向量进行升序排序 index_of_fronts表示排序后的值对应原来的索引

for i = 1 : length(index_of_fronts)

sorted_based_on_front(i,:) = x(index_of_fronts(i),:);%sorted_based_on_front中存放的是x矩阵按照排序等级升序排序后的矩阵

end

current_index = 0;

%% Crowding distance 计算每个个体的拥挤度

for front = 1 : (length(F) - 1)%这里减1是因为代码55行这里，F的最后一个元素为空，这样才能跳出循环。所以一共有length-1个排序等级

distance = 0;

y = [];

previous_index = current_index + 1;

for i = 1 : length(F(front).f)

y(i,:) = sorted_based_on_front(current_index + i,:);%y中存放的是排序等级为front的集合矩阵

end

current_index = current_index + i;%current_index =i

sorted_based_on_objective = [];%存放基于拥挤距离排序的矩阵

for i = 1 : M

[sorted_based_on_objective, index_of_objectives] = ...

sort(y(:,V + i));%按照目标函数值排序

sorted_based_on_objective = [];

for j = 1 : length(index_of_objectives)

sorted_based_on_objective(j,:) = y(index_of_objectives(j),:);% sorted_based_on_objective存放按照目标函数值排序后的x矩阵

end

f_max = ...

sorted_based_on_objective(length(index_of_objectives), V + i);%fmax为目标函数最大值 fmin为目标函数最小值

f_min = sorted_based_on_objective(1, V + i);

y(index_of_objectives(length(index_of_objectives)),M + V + 1 + i)...%对排序后的第一个个体和最后一个个体的距离设为无穷大

= Inf;

y(index_of_objectives(1),M + V + 1 + i) = Inf;

for j = 2 : length(index_of_objectives) - 1%循环集合中除了第一个和最后一个的个体

next_obj = sorted_based_on_objective(j + 1,V + i);

previous_obj = sorted_based_on_objective(j - 1,V + i);

if (f_max - f_min == 0)

y(index_of_objectives(j),M + V + 1 + i) = Inf;

else

y(index_of_objectives(j),M + V + 1 + i) = ...

(next_obj - previous_obj)/(f_max - f_min);

end

end

end

distance = [];

distance(:,1) = zeros(length(F(front).f),1);

for i = 1 : M

distance(:,1) = distance(:,1) + y(:,M + V + 1 + i);

end

y(:,M + V + 2) = distance;

z(previous_index:current_index,:) = y;

end

f = z();%得到的是已经包含等级和拥挤度的种群矩阵 并且已经按等级排序排序

end

function f = replace_chromosome(intermediate_chromosome, M, V,pop)%精英选择策略

V=2*V;

[N, m] = size(intermediate_chromosome);

[temp,index] = sort(intermediate_chromosome(:,M + V + 1));

clear temp m

for i = 1 : N

sorted_chromosome(i,:) = intermediate_chromosome(index(i),:);

end

max_rank = max(intermediate_chromosome(:,M + V + 1));

previous_index = 0;

for i = 1 : max_rank

current_index = max(find(sorted_chromosome(:,M + V + 1) == i));

if current_index > pop

remaining = pop - previous_index;

temp_pop = ...

sorted_chromosome(previous_index + 1 : current_index, :);

[temp_sort,temp_sort_index] = ...

sort(temp_pop(:, M + V + 2),'descend');

for j = 1 : remaining

f(previous_index + j,:) = temp_pop(temp_sort_index(j),:);

end

return;

elseif current_index < pop

f(previous_index + 1 : current_index, :) = ...

sorted_chromosome(previous_index + 1 : current_index, :);

else

f(previous_index + 1 : current_index, :) = ...

sorted_chromosome(previous_index + 1 : current_index, :);

return;

end

previous_index = current_index;

end

end

function f = tournament_selection(chromosome, pool_size, tour_size)

[pop, variables] = size(chromosome);%获得种群的个体数量和决策变量数量

rank = variables - 1;%个体向量中排序值所在位置

distance = variables;%个体向量中拥挤度所在位置

%竞标赛选择法，每次随机选择两个个体，优先选择排序等级高的个体，如果排序等级一样，优选选择拥挤度大的个体

for i = 1 : pool_size

for j = 1 : tour_size

candidate(j) = round(pop*rand(1));%随机选择参赛个体

if candidate(j) == 0

candidate(j) = 1;

end

if j > 1

while ~isempty(find(candidate(1 : j - 1) == candidate(j)))%防止两个参赛个体是同一个

candidate(j) = round(pop*rand(1));

if candidate(j) == 0

candidate(j) = 1;

end

end

end

end

for j = 1 : tour_size% 记录每个参赛者的排序等级 拥挤度

c_obj_rank(j) = chromosome(candidate(j),rank);

c_obj_distance(j) = chromosome(candidate(j),distance);

end

min_candidate = ...

find(c_obj_rank == min(c_obj_rank));%选择排序等级较小的参赛者，find返回该参赛者的索引

if length(min_candidate) ~= 1%如果两个参赛者的排序等级相等 则继续比较拥挤度 优先选择拥挤度大的个体

max_candidate = ...

find(c_obj_distance(min_candidate) == max(c_obj_distance(min_candidate)));

if length(max_candidate) ~= 1

max_candidate = max_candidate(1);

end

f(i,:) = chromosome(candidate(min_candidate(max_candidate)),:);

else

f(i,:) = chromosome(candidate(min_candidate(1)),:);

end

end

end
